# Role of metastasis-associated lung adenocarcinoma transcript-1 (MALAT-1) in pancreatic cancer

**DOI:** 10.1371/journal.pone.0192264

**Published:** 2018-02-01

**Authors:** Yating Cheng, Parisa Imanirad, Indira Jutooru, Erik Hedrick, Un-Ho Jin, Aline Rodrigues Hoffman, Jeann Leal de Araujo, Benjamin Morpurgo, Andrei Golovko, Stephen Safe

**Affiliations:** 1 Department of Veterinary Physiology and Pharmacology, Texas A&M University, College Station, TX, United States of America; 2 Department of Veterinary Pathobiology, Texas A&M University, College Station, TX, United States of America; 3 Texas A&M Institute for Genomic Medicine, Texas A&M University, College Station, TX, United States of America; University of Nebraska Medical Center, UNITED STATES

## Abstract

Metastasis-associated lung adenocarcinoma transcript-1 (MALAT-1) is a long non-coding RNA (lncRNA) that is a negative prognostic factor for patients with pancreatic cancer and several other tumors. In this study, we show that knockdown of MALAT-1 in Panc1 and other pancreatic cancer cell lines decreases cell proliferation, survival and migration. We previously observed similar results for the lncRNAs HOTTIP and HOTAIR in Panc1 cells; however, RNAseq comparison of genes regulated by MALAT-1 shows minimal overlap with HOTTIP/HOTAIR-regulated genes. Analysis of changes in gene expression after MALAT-1 knockdown shows that this lncRNA represses several tumor suppressor-like genes including N-myc downregulated gene-1 (*NDRG-1*), a tumor suppressor in pancreatic cancer that is also corepressed by EZH2 (a PRC2 complex member). We also observed that Specificity proteins Sp1, Sp3 and Sp4 are overexpressed in Panc1 cells and Sp knockdown or treatment with small molecules that decrease Sp proteins expression also decrease MALAT-1 expression. We also generated Kras-overexpressing *p53L/L;LSL-Kras*^*G12D*^*L/+;p48Cre+/-* (p53^L/L^/Kras^G12D^) and *p53L/+;LSLKras*^*G12D*^*L/+;p48Cre+/-* (p53^L/+^/Kras^G12D^) mice which are p53 homo- and heterozygous, respectively. These mice rapidly develop pancreatic ductal adenocarcinoma-like tumors and were crossed with MALAT-1^-/-^ mice. We observed that the loss of one or two MALAT-1 alleles in these Ras overexpressing mice does not significantly affect the time to death; however, the loss of MALAT-1 in the p53^-/+^ (heterozygote) mice slightly increases their lifespan.

## Introduction

Results of high throughput sequencing technologies show that less than 2% of the human genome encodes for proteins, whereas up to 75% of the genome transcribes non-coding RNAs (ncRNAs) which are highly variable in length, function and regulation [1]. MicroRNAs (miRNAs) are 21–23 bp in length and there is evidence showing that miRNAs play important roles in maintaining cellular homeostasis and in diseases such as cancer through their sequence-specific regulation (primarily repression) of genes [2, 3]. There is also evidence that long ncRNAs (lncRNAs) >200 bp play an equally important role in normal cell functions and disease, and estimations from the *Encyclopedia of DNA Element Project Consortium* indicate that the human genome contains up to 16,000 genes encoding 28,000 lncRNA transcripts [1].

Metastasis-associated-lung-adenocarcinoma-transcript-1 (MALAT-1) is a lncRNA that is overexpressed in multiple cancer cell lines and tumors, and MALAT-1 expression in early stage non-small cell lung cancer patients predicted patient survival and metastasis [4]. The expression and prognostic value of MALAT-1 has now been extensively investigated in multiple tumor types showing that high tumor expression of MALAT-1 is a negative prognostic factor for lung, liver, pancreatic, melanoma, cervical, colorectal, gastric, multiple myeloma, clear cell, renal cell, glioma and adrenocortical cancer patients [4–18]. The negative prognosis associated with overexpression of MALAT-1 in tumors correlates with the functions of MALAT-1 in cancer cells. For example, results of knockdown studies in lung cancer cells indicate that loss of MALAT-1 decreased migration/wound healing and injection of MALAT-1-deficient A549 lung cancer cells in mice resulted in significantly decreased formation of lung nodules. In mice bearing A549 cells as xenografts, injection of MALAT-1 antisense oligonucleotides significantly decreased tumor growth [18].

Initial studies reported that MALAT-1 stably localizes to nuclear speckles and interacts with pre-mRNA splicing factors (SR proteins) to modulate gene expression in some cells (e.g. HeLa cells) [19–22]. However, in MALAT-1 knockout mice, formation of nuclear speckles and pre-mRNA splicing were unaffected [23–25], suggesting that pro-oncogenic function of MALAT-1 may be independent of nuclear speckles. MALAT-1 exhibits multiple pro-oncogenic functions in cancer cells and plays a role in cell proliferation, survival, epithelial to mesenchymal transition (EMT), migration and metastasis through diverse mechanisms which include acting as a decoy sponge and direct or indirect interactions with DNA, RNA and protein [26–34]. Recent studies show that MALAT-1 is a negative prognostic factor for pancreatic cancer [7, 11] and results of our studies also demonstrate that MALAT-1 exhibits pro-oncogenic functions in pancreatic cancer cells and the effects are similar to those observed in other tumors. Results of microarray and knockdown studies *in vitro* also suggest a role for MALAT-1 as a scaffold for chromatin modifying complexes. Although loss of MALAT-1 decreased pancreatic tumor growth and metastasis in a mouse orthotopic model [9, 15], the loss of MALAT-1 in highly aggressive *p53L/L;LSL-Kras*^*G12D*^*L/+;p48Cre+/-* and the *p53L/+* heterozygotes only slightly increased survival time in the *p53L/+* heterozygotes.

## Materials and methods

### Cell lines, reagents, and antibodies

Panc28 cells were a generous gift from Dr. Paul Chiao (University of Texas MD Anderson Cancer Center, Houston, TX); L3.6pL cells were kindly provided by I. J. Fidler (University of Texas MD Anderson Cancer Center); and HPDE cells were provided by Dr. Ming Sound Tsao (Ontario Cancer Institute, Toronto, Canada). Panc1, ASPC1, BxPC3 and MiaPaCa2 cells were obtained from the American Type Culture Collection (Manassas, VA). Panc1, MiaPaCa2 and L3.6pL cells were authenticated on April 29, 2016 by Biosynthesis (Lewisville, TX). Panc1, L3.6pL, Panc28 and MiaPaCa2 cells were maintained in Dulbecco's modified Eagle medium (DMEM) (GenDEPOT, Barker, TX, USA) supplemented with 10% fetal bovine serum (FBS). BxPC3 and ASPC1 cells were maintained in RPMI-1640 medium (GenDEPOT, Barker, TX, USA) supplemented with 10% FBS. Cells were grown in 150-cm^2^ culture plates in an air-CO_2_ (95:5) atmosphere at 37°C and passaged approximately every 3 to 5 days. Cleaved PARP (D214), cleaved caspase-7 (Asp198) (D6H1), cleaved caspase-9 (Asp330) (D2D4), and GAPDH antibodies were purchased from Cell Signaling Technology (Danvers, MA). Sp3 and Sp4 antibodies were purchased from Santa Cruz Biotech (Santa Cruz, CA, USA) and an Sp1 antibody was purchased from Abcam (Cambridge, MA, USA). β-Actin (A1978) antibody was obtained from Sigma-Aldrich. 3-(4,5-dimethylthiazol-2-yl)-2,5-diphenyltetrazolium bromide (MTT); glutathione was purchased from ThermoFisher Scientific (Waltham, MA, USA). Chemiluminescence reagents (Immobilon Western) for Western blot imaging were purchased from Millipore (Billerica, MA). Lipofecatmine 2000 was purchased from Invitrogen (Carlsbad, CA).

### RNA interference

Pancreatic cancer cells were seeded (1x10^5^ per well) in 6-well plates in DMEM medium supplemented with 2.5% FBS and left to attach for 1 day. Knockdown by RNA interference (RNAi) with siCtrl as a control was performed using lipofecatmine 2000 transfection reagent as per the manufacturer's instructions. Primers for siRNA studies are listed in S1 Table.

### Real time-PCR

Total RNA was isolated using Zymo Quick RNA MiniPrep Kit (Zymo Research, Irvine, CA) according to the manufacturer's protocol. RNA was eluted with 35 μl of RNase-free water and stored at -80°C. Real-time (RT)-PCR was carried out using iTaq Universal SYBR Green One-step Kit (Bio-Rad, Hercules, CA). Primers for human and mouse genes are summarized in S2 and S3 Tables.

### Western blot analysis

Seventy-two hours after siRNA transfection, cells were collected using high-salt buffer (50 mM HEPES, 0.5 mol/l NaCl, 1.5 mM MgCl_2_, 1 mM EGTA, 10% glycerol, and 1% Triton-X-100) and 10 μl/ml Protease Inhibitor Cocktail (Sigma-Aldrich). Protein lysates were incubated for 5 min at 95°C before electrophoresis and then separated on 10% SDS-polyacrylamide gel electrophoresis 120 V for 2 to 3 hr. Proteins were transferred onto polyvinylidene difluoride membranes by wet electroblotting in a buffer containing 25 mM Tris, 192 mM glycine, and 20% methanol for 1.5 hr at 900 mA. Membranes were then blocked for 30 min with specific antibodies. Detection of specific proteins was performed using Chemiluminescence and then exposed to Kodak image station 4000 mm Pro (Carestream Health, Rochester, NY).

### Cell proliferation assays

#### Cell counting

Pancreatic cancer cells were seeded in 12-well plates, and 72 hr after siRNA transfection, cells were trypsinized and counted using a Coulter Z1 cell counter (Beckman Coulter, Fullerton, CA).

#### MTT assay

Pancreatic cells were seed into a 96-well plate and 72 hr after siRNA transfection, medium was removed, and MTT solution diluted in PBS was added to cell cultures. After 2 hr incubation, the medium was aspirated and washed with PBS. Dimethyl sulfoxide (DMSO) was added and incubate at 37° for 10 min and absorbance was measured at 570 nM.

### Apoptosis and cell cycle analysis assays

For cell cycle analysis, 48 hr after transfection, cells were stained with propidium iodide solution (50 μg/ml) and were analyzed by fluorescence-activated cell sorter (FACS). Apoptosis was detected using Alexa Fluor 488 Annexin V/Dead Cell apoptosis kit followed by FACS analysis according to the manufacturer's protocol. We routinely measure cleaved PARP in western blots for apoptosis markers and observe that the expression inversely correlates with full length PARP.

### Migration and invasion assays

#### Transwell migration/invasion

Pancreatic cancer cells were first transfected with siRNAs for 48 hr, then added to the upper chamber of a transwell chamber (with or without matrigel) in duplicate and allowed to migrate/invade into the lower chamber containing DMEM media with 20% FBS by incubating for 24 hr. Cells migrating/invading to the outer side of the upper chamber were fixed, stained and counted.

#### Scratch assay

Cells were first seed in 6-well plates for 24 hr, and then a scratch through the central axis of the plate was gently made using a sterile pipette tip. Cells were transfected with siRNAs, and media was changed after 6 hr. Migration of the cells into the scratch was observed after 24, 48, and 72 hr. At the 0 hr time point, the "scratched" lane was completely clear with no migrated cells. Ibidi assay: Pancreatic cancer cells were first transfected with siRNAs for 24 hr, then seeded in the silicone cell culture inserts (Ibidi, Martinsried, Germany) which are attached to culture plates. Inserts were removed with tweezers and cells were rinsed with PBS and new medium was added into the cell after 24 hr and movement of cells into the middle gap was determined.

### Microarray analysis

Total RNA was isolated using Zymo Quick RNA MiniPrep Kit (Zymo Research, Irvine, CA) according to the manufacturer's protocol. RNA was eluted with 35 μl of RNase-free water and stored at -80°C. The total RNA was quantified by using a NanoDrop ND-1000 spectrophotometer (NanoDrop Technology). The total RNA samples with adequate RNA quality index (>7) were used for microarray analysis; 700 ng of total RNA was used for labeling and hybridization on Human HT-12 v4 expression beadchip (Illumina, Inc.) according to the manufacturer's protocols. After the beadchips were scanned with a BeadArray Reader (Illumina), the microarray data were normalized using the quantile normalization method in the Linear Models for Microarray Data (LIMMA) package in the R language (http://www.r-project.org). BRB-Array Tools were primarily used for statistical analysis of gene expression data, and the Student’s *t* test was applied to identify the genes significantly different between 2 groups when compared. Function and pathway analysis of differentially regulated genes was determined using Ingenuity Pathway Analysis (IPA) database (Invitrogen, Carlsbad, CA).

### RNA sequencing analysis

Total RNA was isolated using Zymo Quick RNA MiniPrep Kit (Zymo Research, Irvine, CA) according to the manufacturer's protocol. RNA was eluted with 35 μl of RNase-free water and stored at -80°C. The total RNA was quantified by using a Nanodrop ND-1000 spectrophotometer (NanoDrop Technology). RNA sequencing was carried out in the genomics and bioinformatics service at Texas A&M University and analyzed using sequencing-pipeline developed by Dr. Robert Chapkin, Texas A&M University. Functional and pathway analysis of differentially regulated genes was determined using Ingenuity Pathway Analysis (IPA) database (Invitrogen, Carlsbad, CA).

### Animal studies and production of Malat-1 KO mice

The Texas A&M University Institutional Animal Care and Use Committee approved all of the animal procedures (Animal Use Protocols 2014–0246 and 2016–0174), which is in compliance with the standards outlined in the guide for the Care and Use of Laboratory Animals. A total of 48 animals have been monitored during the period of 2 years and 9 months, 23 of which have been euthanized and 25 found dead or went missing. In many instances the disease progressed very quickly and animals were found dead before displaying signs of sickness. On other occasions, mice were euthanized upon display of the signs of sickness (weight loss, hunched posture, scruffy hair). Animals were monitored daily or at least once every 2–3 days during weekends and holidays. Animal care staff was trained to monitor health and well-being of the experimental animals and inform research staff of any changes in animal conditions. All animal work was performed under IACUC-approved animal use protocol drafted specifically to perform analysis of various phenotypic outcomes. Hunched posture, scruffy hair, dehydration, visible weight loss, lethargy—all as compared to healthy wt littermates. Cages with experimental animals were clearly marked with signs for husbandry personnel to report any signs of animals in distress or mortalities. Animals carrying disease-causing alleles were observed on daily basis and any signs of possible phenotype were recorded in a spreadsheet.

Malat-1 knockout mice were generated using a gene-trapping technique [35]. Mice (strain C57BL/6) were cloned from an ES cell line (IST14461G11; Texas A&M Institute for Genomic Medicine, TIGM). The ES cell clone contained a retroviral insertion in the Malat-1 gene identified from the TIGM gene trap database, and was microinjected into C57BL/6 host blastocysts to generate germline chimeras using standard procedures [36]. The retroviral OmniBank Vector 74 contained a splice acceptor sequence (SA) followed by a 5' selectable marker neomycin resistance gene, for identification of successful gene trap events followed by a polyadenylation signal (pA). Insertion of the retroviral vector into the Malat-1 gene led to the splicing of the endogenous upstream exons into this cassette to produce a fusion transcript and terminate expression of the RNA downstream. Chimeric males were bred to C57BL/6 females for germline transmission of the mutant Malat-1 allele. Ablation of Malat-1 expression in homozygous mice was confirmed by RT-PCR.

### Animal pathology

A total of 17 mice were euthanized with carbon dioxide (CO_2_). Of these, one was genotype p53L/L LSL-KrasG12D L/+ P48Cre +/-; two were genotype Malat-1 -/- p53L/L LSL-KrasG12D L/+ P48Cre +/-; nine were genotype p53L/+ LSL-KrasG12D L/+ P48Cre+/-; three were genotype Malat-1+/- p53L/+ LSL-KrasG12D L/+ P48Cre+/-; and two were genotype Malat-1-/- p53L/+ LSL-KrasG12D L/+ P48Cre+/-. The following tissues were harvested from all these mice and fixed in 10% neutral buffered formalin: heart, lungs, diaphragm, pancreas, liver, kidney, spleen, stomach, small intestine, large intestine, testis/ovaries and brain. Fixed tissues were processed for routine histology and paraffin embedded. Histological sections were stained with hematoxylin and eosin, and examined by a board certified pathologist (ARH).

### Statistical analysis

Statistical significance of differences between the treatment groups was determined by an analysis of variance and/or Student's *t* test, and levels of probability were noted. At least 3 repeated experiments were determined for each data points and results are expressed as means ± SD.

## Results

### Expression and pro-oncogenic functions of MALAT-1 in pancreatic cancer cells

**Fig 1:**
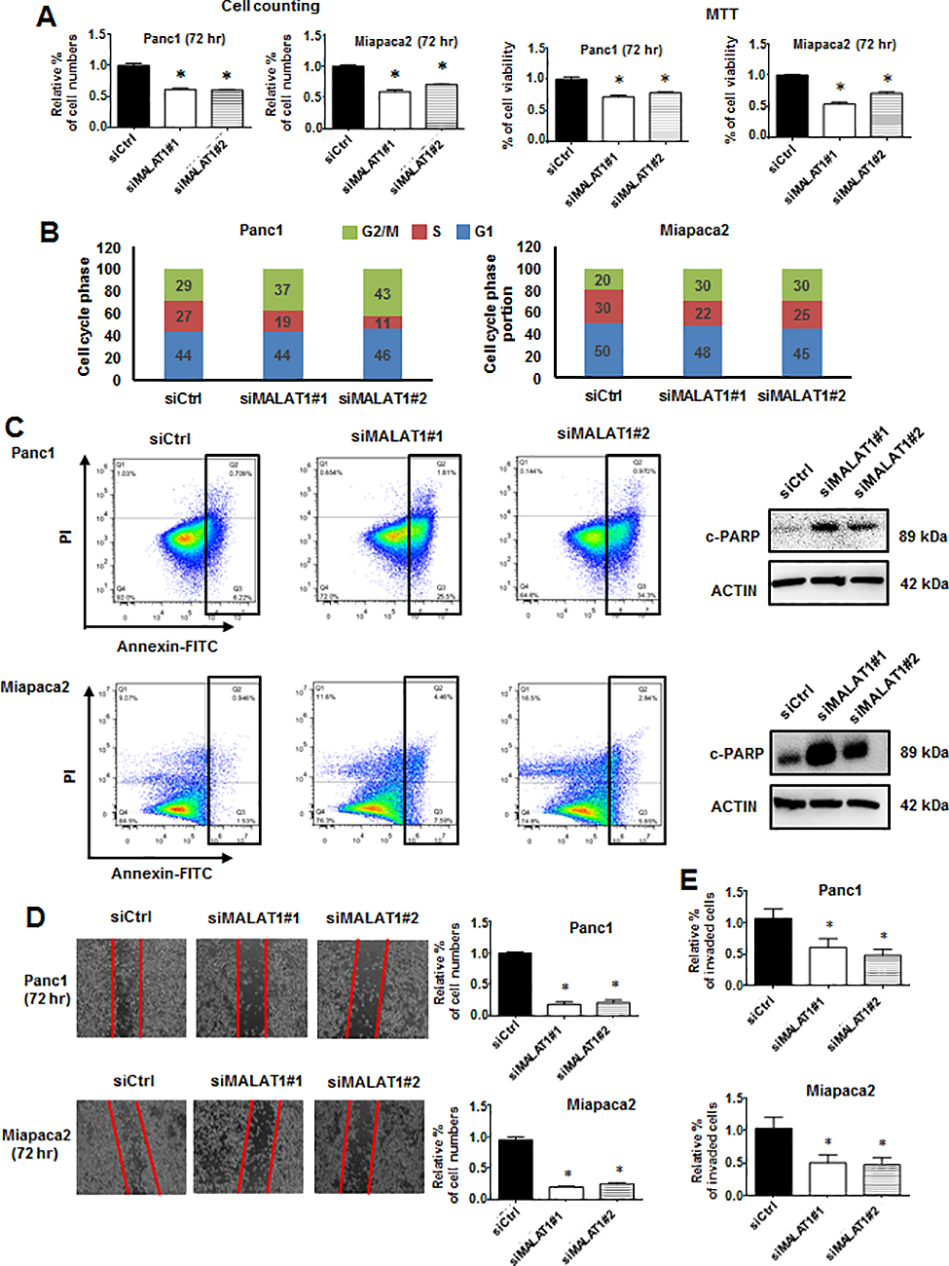
Effects of MALAT-1 in pancreatic cell proliferation, cell cycle, apoptosis, migration and invasion. (A) MALAT-1 knockdown by RNAi in Panc1, MiaPaCa2 inhibited cell growth. (B) The effect of siMALAT-1 (knockdown) on cell cycle progression in Panc1 and MiaPaCa2 cells was determined by FACS analysis. (C) The apoptotic cells were quantified using FACS analysis and induction of PARP cleavage was determined by western blot analysis. MALAT-1 knockdown reduced cell migration (D) and cell invasion (E) as determined by scratch assay and Boyden chamber assay, respectively. Significant (p<0.05) changes are indicated (*).

Previous studies showed that MALAT-1 is overexpressed in pancreatic cancer cell lines and tumors compared to non-transformed pancreatic cells/tissue [15, 16]. We also observed high expression of MALAT-1 in pancreatic cancer cells (S1A Fig); knockdown of MALAT-1 (siMALAT-1) in Panc1 and MiaPaCa2 cells decreased cell proliferation and induced G2/M arrest (Fig 1A and 1B) and induced apoptosis as indicated by increased Annexin V staining and expression of cleaved PARP (Fig 1C). Moreover, knockdown of MALAT-1 also decreased cell migration and invasion in scratch and Boyden chamber assays (Fig 1D and 1E), and decreased migration was also observed in ibidi and Boyden chamber assays (S1B and S1C Fig). These results confirm that MALAT-1 plays a role in pancreatic cancer cell proliferation, survival and migration/invasion.

### Analysis of MALAT-1 regulated gene expression in Panc1 cells

**Fig 2:**
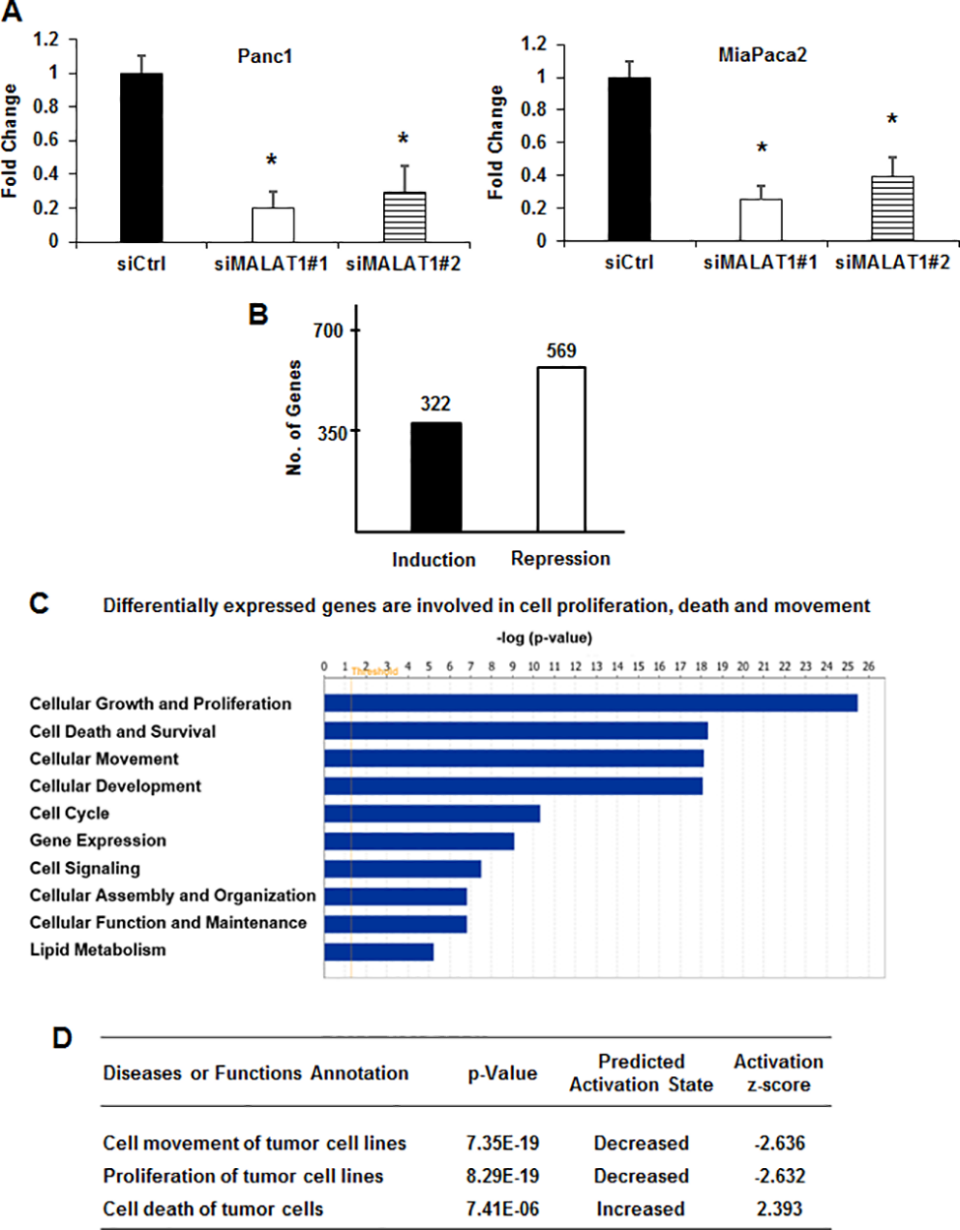
Gene regulation by MALAT-1. (A) Panc1 cells were transfected with oligonucleotides (siMALAT-1#1/siMALAT-1#2) and expression of MALAT-1 was determined by real time PCR. (B) Panc1 cells were transfected with siMALAT-1 or siCtrl and gene expression was analyzed using Human HT-12 v4 expression beadchip (Illumina, Inc.) array. (C) The effects of siMALAT-1 on different function categories and the predicted activation state of cell proliferation, death and movement after HOTTIP knockdown were determined by IPA. (D) Pathway analysis. Causal IPA was used to analyze the p-values and Z-scores for cell movement proliferation and cell death after MALAT-1 knockdown by RNA interference.

**Fig 3:**
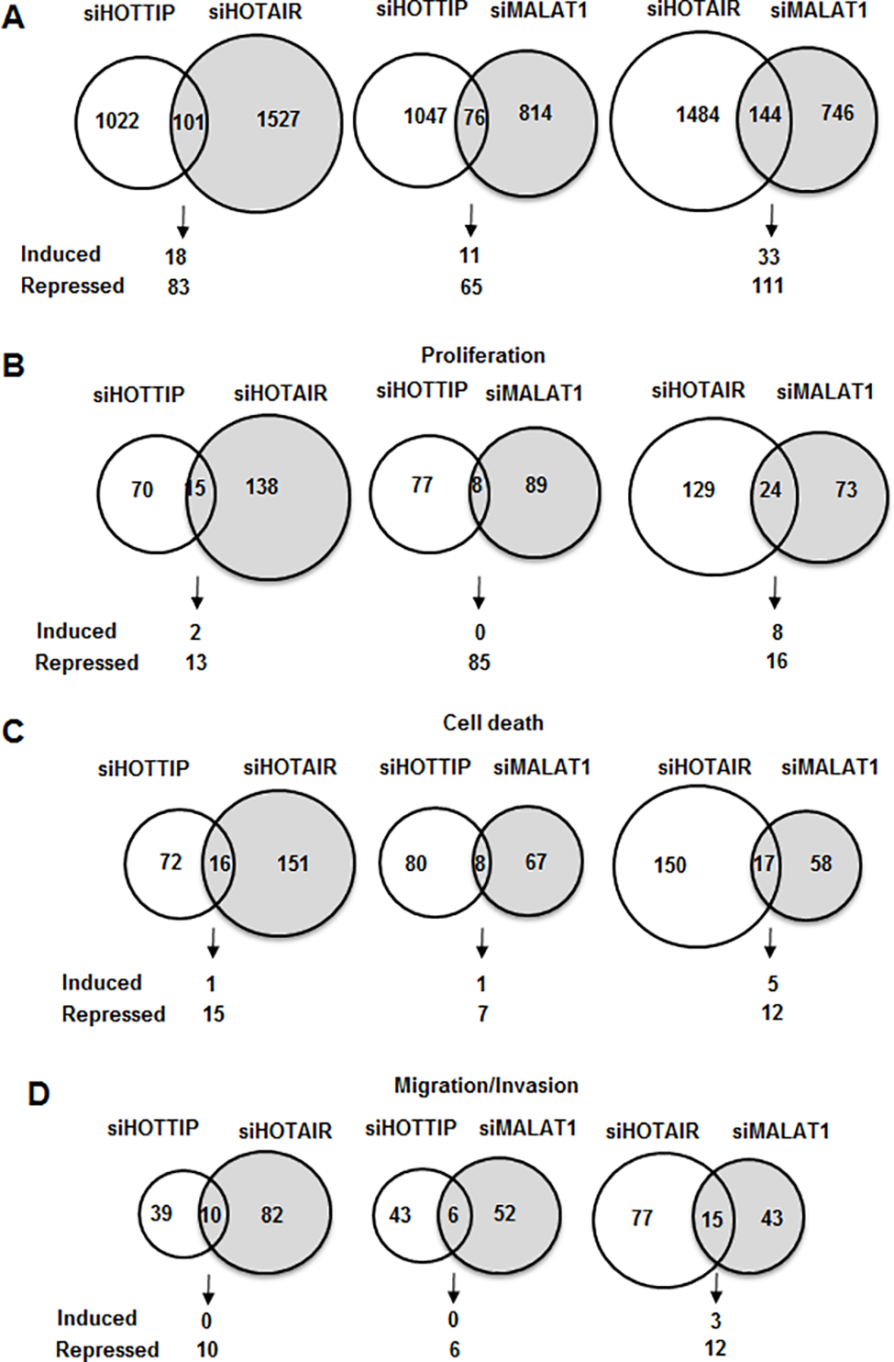
Panc1 cells were transfected with siHOTTIP, siHOTAIR or siMALAT-1, and changes in gene expression were determined using human HT-12 V4 expression bead chip arrays. The overlap of total genes (A), proliferation inhibition (B), cell death (C) and inhibition of migration/invasion (D) genes coregulated by HOTTIP/HOTAIR, HOTTIP/MALAT-1 and HOTAIR/MALAT-1 was determined by IPA.

Knockdown of MALAT-1 by RNAi (Fig 2A) or treatment with a non-specific control (siCtl) oligonucleotide in Panc1 cells resulted in the induction of 352 and repression of 611 genes (Fig 2B) as determined by RNAseq. Further analysis of these genes by Ingenuity Pathway Analysis demonstrated that MALAT-1-regulated genes are involved in multiple functions (Fig 2C). Many of the MALAT-1-regulated genes were associated with cell growth and proliferation, cell death and survival, and cell movement (motility, migration and invasion) and these correlated with functional responses observed after MALAT-1 knockdown (Fig 1). Fig 2D summarizes analysis of the overall changes in gene expression after MALAT-1 knockdown using causal Ingenuity Pathway Analysis which is a quantitative method that integrates both changes in gene expression and pathways to predict biologic function. The low *p* values and activation scores (> 2.0 or < -2.0) obtained from causal IPA strongly predicted that loss of MALAT-1 was associated with decreased cell movement and proliferation and increased cell death, and this analysis correlated with results of functional studies (Fig 1). Previous studies in this laboratory showed that like MALAT-1, the lncRNAs HOTAIR and HOTTIP also regulate cell proliferation, survival and movement in Panc1 pancreatic cancer cells [37, 38], and Fig 3A shows the overlap of total changes in gene expression observed for MALAT-1, HOTTAIR and HOTTIP. Based on the total number of genes regulated by MALAT-1 (963), HOTAIR (1628) and HOTTIP (1125), the common genes coregulated by MALAT-1/HOTTIP and MALAT-1/HOTAIR were only 8.5 and 16.1%, respectively, suggesting that the common pro-oncogenic responses regulated by the three lncRNAs are primarily due to different sets of genes. Venn diagram analysis of the overlap in gene expression associated with cell proliferation, cell death and migration/invasion by MALAT-1, HOTAIR and HOTTIP (Fig 3B–3D) shows that there was minimal gene overlap (<10%), further demonstrating that the lncRNA-regulation of these responses was primarily due to different sets of genes.

**Fig 4:**
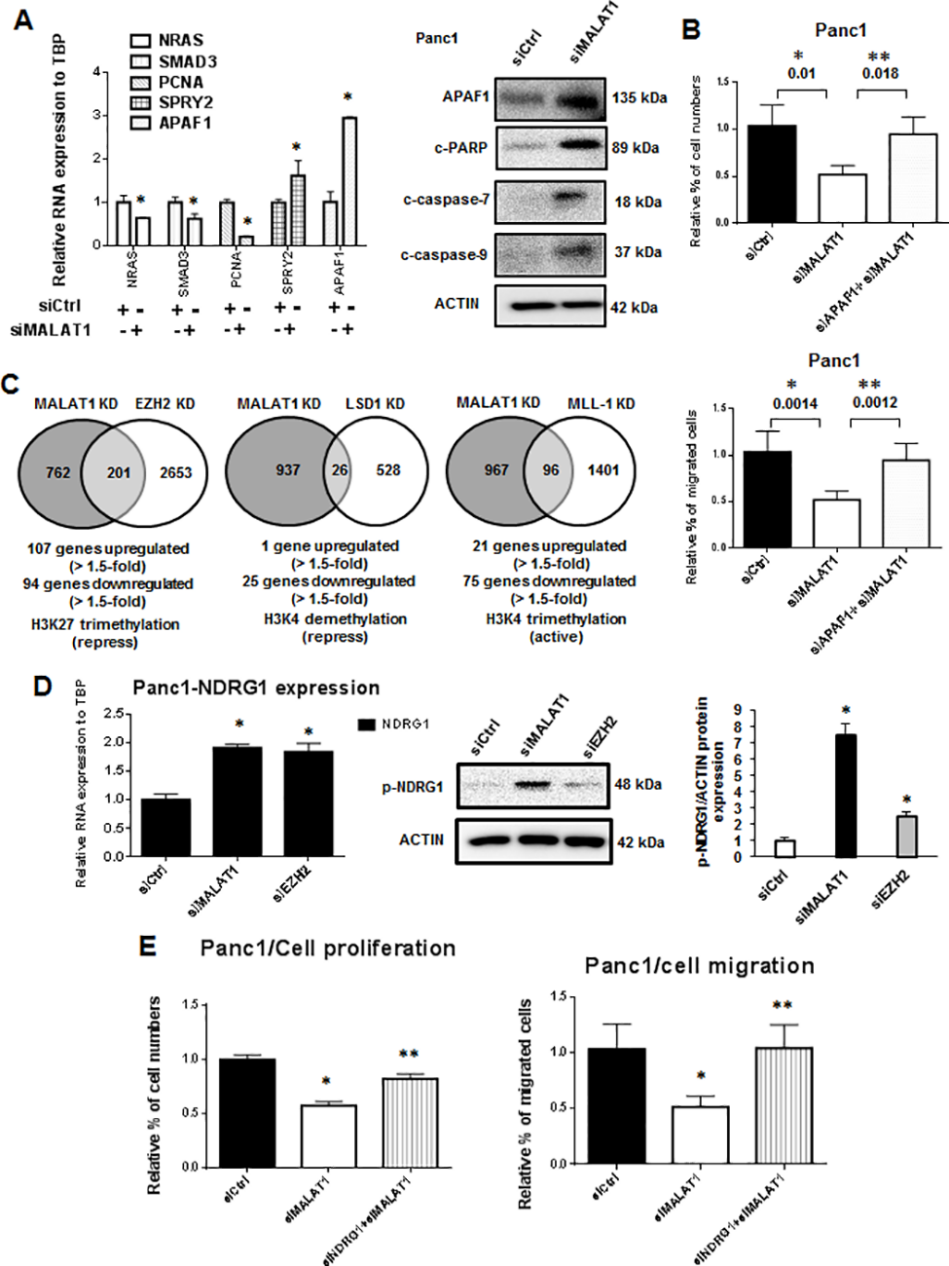
MALAT-1 regulated genes and interactions between MALAT-1 and EZH2 on gene expression in Panc1 cells. (A) Cells were transfected with siMALAT-1 or siCtrl. Genes regulated by MALAT-1 were analyzed by real time PCR and the expression of apoptosis associated proteins was analyzed by western blot. (B) Panc1 cells were transfected with siMALAT-1, siMALAT-1+siAPAF1 or siCtrl, and effects on cell growth and migration were determined. (C) Panc1 cells were transfected with siMALAT-1, siEZH2, siLSD1, siMLL1 or siCtrl, analyzed by microarrays for changes in gene expression. (D) Cells were transfected with siMALAT-1, siEZH2, or siCtrl, and the mRNA and protein expression of NDRG1 were determined by real time PCR and western blot. (E) Panc1 cells were transfected with siMALAT-1, siMALAT-1+siNDRG1 or siCtrl and effects on cell growth and migration were determined. Significant (p<0.05) changes are indicated (*) or (**).

Fig 4A illustrates induction and repression of potentially functional genes in pancreatic cancer observed after MALAT-1 knockdown in Panc1 cells as determined by real time PCR. One of these genes, apoptotic protease activating factor 1 (APAF1), is a key protein component of the apoptosome, and knockdown of MALAT-1 induced expression of APAF1 protein and activation (cleavage) of caspases 7 and 9 and PARP in Panc1 cells (Fig 4A). This suggests that the pro-apoptotic activity observed after MALAT-1 knockdown was due, in part, to induction of APAF1. Fig 4B shows that in Panc1 cells transfected with siMALAT-1, there was a decrease in cell migration and this was partially reversed by knocking down APAF-1 (induced by siMALAT-1), suggesting that MALAT-1-mediated induction of APAF-1 may also play a role in Panc1 cell growth and migration.

We also used RNAi coupled with analysis of array data to investigate the overlap of MALAT-1 with EZH2-, LSD1- and MLL-1-regulated genes to identify genes coregulated by PRC2, REST/coREST and MLL-1 chromatin modifying complexes, respectively. There was an overlap of genes coregulated by MALAT-1 and EZH2, LSD1 and MLL-1 with the highest gene overlap observed with EZH2 (a component of the PRC2 complex) in which 107 common genes were upregulated after Panc1 cells were transfected with siMALAT-1 and siEZH2 (Fig 4C). N-myc downregulated gene-1 (*NDRG1*) which exhibits tumor suppressor-like activity in pancreatic cancer is an example of a gene coregulated by MALAT-1/EZHZ [39–41]. Knockdown of MALAT-1 or EZH2 by RNAi in Panc1 cells induced expression of NDRG1 mRNA as determined by real time PCR (Fig 4D), and western blot analysis showed these treatments also increased NDRG1 protein and the effects were more pronounced after MALAT-1 knockdown. The functional role of MALAT-1-dependent suppression of NDRG1 was further investigated in Panc1 cells transfected with siMALAT-1 which decreased cell proliferation and migration (Fig 4E) and this was partially reversed by cotransfection with siNDRG1. Thus, NDRG1 suppression by MALAT-1 also plays a role in the pro-oncogenic functions of this lncRNA associated with cell proliferation and migration.

### MALAT-1 is an Sp-regulated gene

**Fig 5:**
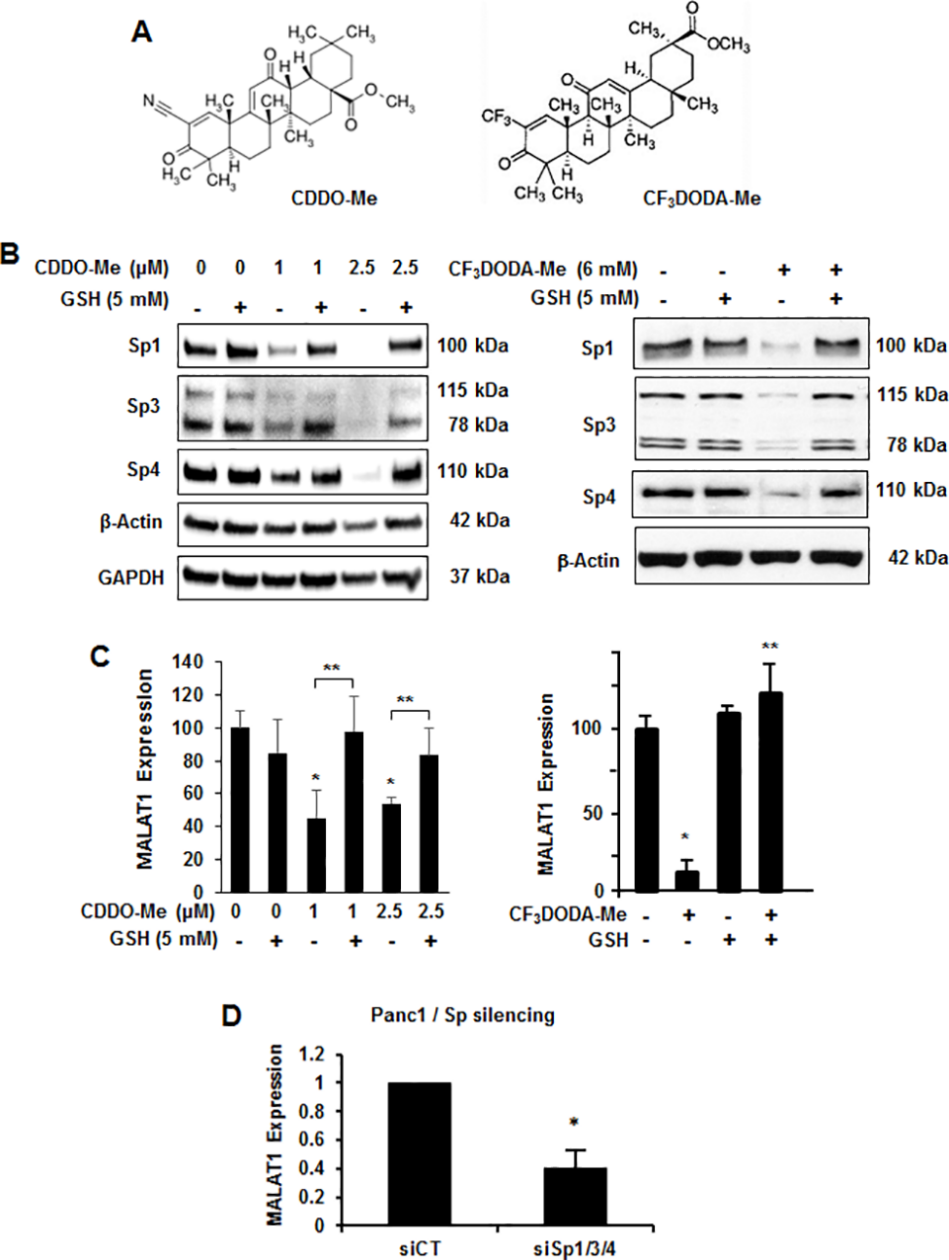
**(A) The structure of CDDO-Me and CF**_**3**_**DODA-Me.** Panc1 cells were treated with different concentrations of CDDO-Me or in combination of GSH, CF_3_DODA-Me or in combination with GSH, and the changes of different proteins (B) and MALAT-1 expression (C) were determined by western blot and real time PCR, respectively. (D) Panc1 cells were transfected with siSp1/3/4 or siCtrl, and the MALAT-1 RNA expression was determined by real time PCR. Significant (p<0.05) changes are indicated (*) or (**).

The transcription factors Sp1, Sp3 and Sp4 are overexpressed in pancreatic cancer cells and like MALAT-1, knockdown of Sp1, Sp3 and Sp4 or Sp1/Sp3/Sp4 (combined) results in decreased cell growth, induces apoptosis, and decreases migration [42–45]. A recent report showed that MALAT-1 is an Sp1-regulated gene [13] and we therefore determined if ROS-inducing anticancer agents that downregulate Sp1, Sp3 and Sp4 in pancreatic and other cancer cell lines also decrease MALAT-1 expression. Treatment of Panc1 cells with two triterpenoid ROS inducers, methyl-2-cyano-3,12-dioxooleana-1,9-dien-28-oate (CDDO-Me, bardoxolone-methyl) and methyl-2-trifluoromethyl-3,11-dioxo-18β-olean-1,12-dien-30-oate (CF_3_DODA-Me) (Fig 5A), decreased expression of Sp1, Sp3 and Sp4, and these effects were reversed in Panc1 cells cotreated with the antioxidant GSH (Fig 5B). These results are consistent with previous reports on ROS inducers that downregulate Sp proteins in pancreatic and other cancer cell lines [42–45]. Treatment of Panc1 cells with the two triterpenoids alone also decreased MALAT-1 expression and cotreatment with GSH also attenuated this response (Fig 5C). Moreover, silencing Sp1, Sp3 and Sp4 combined (siSp1/3/4) by RNA interference also decreased MALAT-1 expression (Fig 5D), confirming that MALAT-1 is an Sp-regulated gene as previously reported [13].

### *In vivo* studies on effects of MALAT-1 loss in genetic models of pancreatic cancer

**Fig 6:**
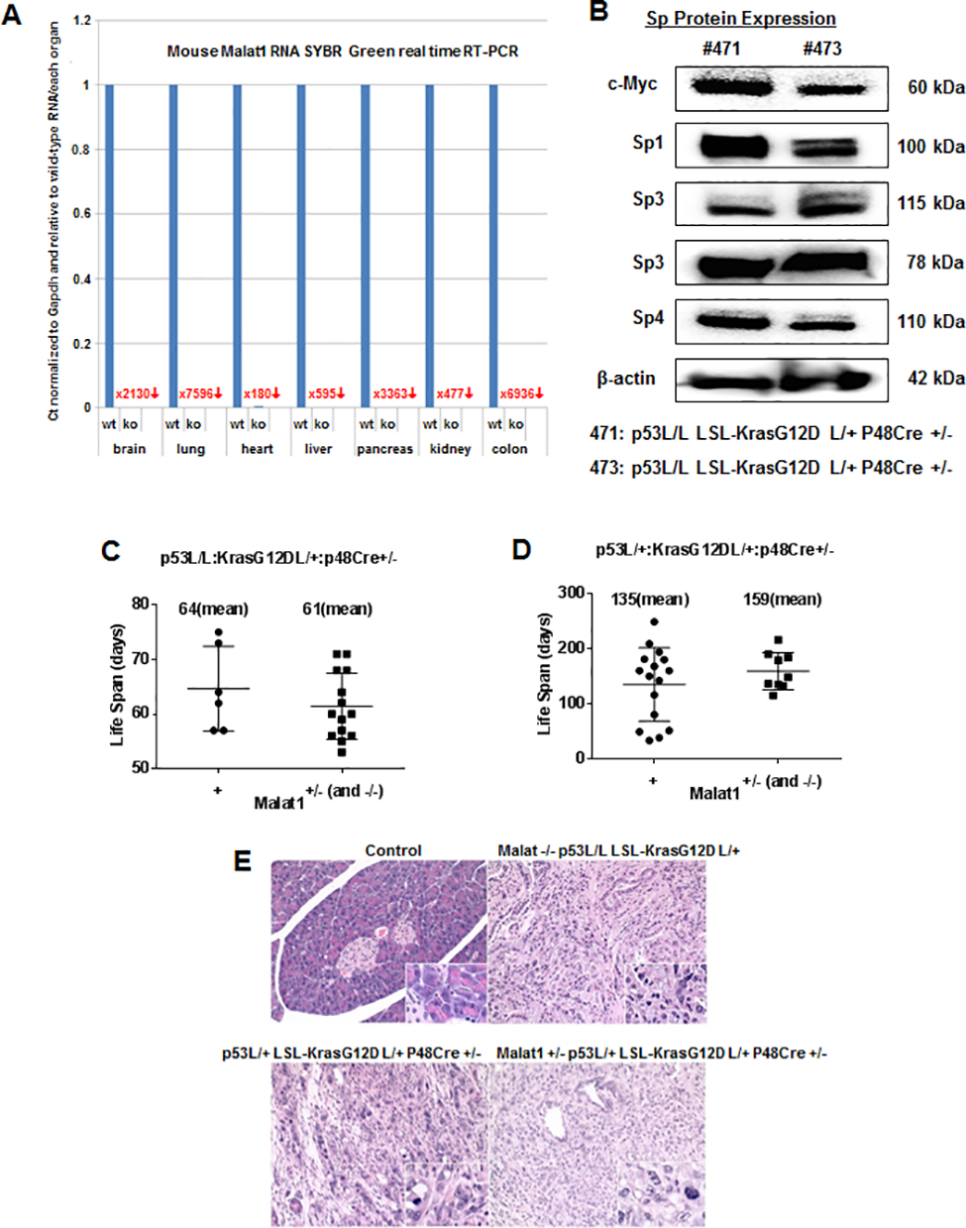
**(A) Fold-change in MALAT-1 gene expression in knockout mice as compared to the wild type.** (B) Sp1, Sp3, Sp4 and c-Myc expression in homozygous floxed p53/Kras^GD12^ mice. (C) Survival of homozygous floxed p53L/L: Kras^GD12^:p48Cre+/- mice expression MALAT-1 (+) or with loss of MALAT-1 (-/+). (D) Survival of heterozygous floxed p53L/+: Kras^GD12^:p48Cre+/- mice expression MALAT-1 (+) or with loss of MALAT-1 (-/+). (E) Histology analysis of tumor samples from different strains of mice; images were 200X and 600X (for corner inserts). p-Values for significant differences in (C) and (D) were 0.39 and 0.26, respectively.

MALAT-1^-/-^ mice have previously been reported [23–25] and were also generated in our laboratory using a gene trapping technique [35], and results in Fig 6A show that real time PCR analysis did not detect MALAT-1 expression in multiple tissues of MALAT-1^-/-^ mice. Using founder mice provided by the DePinho laboratory [46, 47], we generated *p53L/L;LSL-Kras*^*G12D*^*L/+;p48Cre+/-* (p53^L/L^/Kras^G12D^) and *p53L/+;LSLKras*^*G12D*^*L/+;p48Cre+/-* (p53^L/+^/Kras^G12D^) mice which are p53 homo- and heterozygous, respectively. We observed high expression of Sp1, Sp3 and Sp4 in pancreatic tumors from these mice (Fig 6B), whereas low expression of these proteins has been reported in normal mouse tissues [48]. These transgenic mice rapidly develop tumors and typically present with adverse symptoms 12 to 24 hr prior to tumor-induced lethality. Results in Fig 6C and 6D show that p53 heterozygous mice live longer than the corresponding p53^-/-^ mice; moreover in these two mouse models, the loss of one or two MALAT-1 alleles does not significantly affect the time to death, although the results suggest that the loss of MALAT-1 in the p53^-/+^ mice results in an increased (not significant) lifespan. RNAseq was used to determine differences in pancreatic cancer gene expression in Ras overexpressing/p53^+/-^ mice ± MALAT-1 expression and we observed that >1000 genes were differentially expressed. A comparison of the Panc1 gene analysis (Fig 2) vs. the *in vivo* indicates that 50 genes were commonly altered after loss of MALAT-1 (see S4 Table) and this included NDRG1. Most experimental mice presented with a high grade ductular adenocarcinoma (DAC) (Fig 6E), independent of their phenotype which was composed of infiltrative and unencapsulated neoplasms arranged in variably sized ducts separated by abundant fibrovascular stroma. Neoplastic cells were cuboidal, with moderate amounts of eosinophilic cytoplasm and round nuclei. Anisocytosis and anisokryosis were marked with high mitotic activity. There were multifocal areas of necrosis within the neoplasm and in some cases the neoplasm infiltrated adjacent lymph nodes, the muscularis of the stomach and small intestine and rarely the diaphragm. Several other pathological findings were observed; however, these were not consistent with specific changes in p53 (homo- and heterozygote) or MALAT-1. Thus, the loss of MALAT-1 had minimal effects on pancreatic tumors in transgenic mice where there is activation of Kras and a loss of p53; however, in p53 heterozygote mice, there was an increase (not significant) in survival after loss of MALAT-1 (one or two alleles).

## Discussion

MALAT-1 expression in many tumors is a negative prognostic factor for patients, and results obtained in this study and previous reports confirm that MALAT-1 is a pro-oncogenic factor for pancreatic cancer (Fig 1). We have previously reported that the lncRNAs HOTAIR and HOTTIP regulate expression of genes associated with pancreatic cancer cell proliferation, survival and migration [37, 38]. A comparison of genes regulated by MALAT-1 and HOTTIP and HOTAIR in Panc1 cells indicates <6% overlap in coregulated genes, and similar results were observed after comparing specific gene sets associated with proliferation, survival and migration (Fig 3B–3D). APAF1 is an example of a gene regulated (suppressed) by MALAT-1 and not HOTAIR or HOTTIP, and APAF1 induction after MALAT-1 knockdown plays an important role in activating apoptosis and other pathways in Panc1 cells (Fig 4B). Interestingly, although MALAT-1, HOTAIR and HOTTIP exhibit similar functional pro-oncogenic activities in pancreatic cancer cells (Fig 1), the targeted (RNAi) loss of any one of these lncRNAs cannot be rescued by the other two lncRNAs and this supports the array data showing their regulation of different sets of genes, and we are currently investigating the functions of key genes differentially regulated by MALAT-1, HOTTIP and HOTAIR.

Several genetic mouse models of pancreatic cancer have been developed [49] and in this study, we used mice with p53 mutations and overexpression of activated Kras in the pancreas since they rapidly develop pancreatic tumors that resemble human PDAC [47]. However, p53 heterozygotes survive longer than homozygous mice and the loss of MALAT-1 only slightly extends the survival of the latter (not statistically significant) but not the former mice (Fig 6C and 6D). Thus, the loss of MALAT-1 in the transgenic mice driven by Kras expression and p53 deletion (-/- or +/-) had only a minimal impact on this aggressive tumor model, whereas the loss of MALAT-1 in an orthotopic and xenograft mouse models of pancreatic cancer decreased tumor growth and invasion [9, 21]. Since Ras activation is observed in most pancreatic tumors, it is unlikely that drugs targeting MALAT-1 alone would be effective. Liby and coworkers [50] reported that treatment of a similar transgenic mouse model [*LSL-Kras*^*G12D/+*^*;LSL-Trp53*^*R127H/+*^*;Pdx-1-Cre* (KPC)] with CDDO-Me (15 mg/kg body weight) increased survival from 20.5 ± 0.9 (control) to 24.2 ± 2.7 weeks. Moreover, they also reported that CDDO-Me induced ROS in pancreatic cancer cells [50]. This was consistent with studies in this laboratory showing that CDDO-Me induced ROS and ROS-dependent downregulation of Sp1, Sp3 and Sp4 and pro-oncogenic Sp-regulated genes and has been observed with other ROS-inducing anticancer agents in pancreatic cancer cells [42–44]. Results illustrated in Fig 5 show that the ROS-inducing triterpenoids CDDO-Me and CF_3_DODA-Me induce ROS-dependent downregulation of Sp1, Sp3 and Sp4 in Panc1 cells (Fig 5B) and also MALAT-1 (Fig 5C), which is an Sp-regulated gene (Fig 5D). Thus, since MALAT-1 is an Sp-regulated gene, ROS-inducing anticancer agents that target Sp1, Sp3 and Sp4 also decrease MALAT-1 expression and downstream pro-oncogenic effects of MALAT-1. High levels of Sp1, Sp3 and Sp4 protein are expressed in transgenic mice (Fig 6B); however, the effectiveness of CDDO-Me in extending the life of these mice [50] is due not only to decreased MALAT-1 expression but also several pro-oncogenic Sp-regulated genes including bcl-2, survivin, epidermal growth factor receptor, other receptor tyrosine kinases and p65(NFκB) [42–45].

Thus, results of this study confirm the pro-oncogenic activity of MALAT-1 in pancreatic cancer cells and demonstrate that this activity is distinct from that previously observed for HOTAIR and HOTTIP in Panc1 cells. Loss of MALAT-1 in the Ras overexpressing mice prolonged life only in the p53 heterozygote and not homozygote mice; however, it is apparent in the p53/Kras mouse models that MALAT-1 does not significantly alter the progression of this disease and overall lifespan. Current studies are focused on using genetic mouse models for pancreatic cancer in which both MALAT-1 and Sp transcription factors can be deleted (tissue-specific) or specifically targeted to confirm this important role of these genes in tumor development and growth and thereby demonstrate the utility of ROS-inducing anticancer agents such as CF_3_DODA-Me and CDDO-Me for treating this disease.

## Supporting information

S1 Table Primers for siRNA studies. (DOCX)

S2 Table Human primers used for real time-PCR. (DOCX)

S3 Table Mouse primers used for real time-PCR. (DOCX)

S4 Table The comparison of differentially expressed gene in Panc1 cells and Malat1 transgenic mouse tumors. Mouse: 1251 genes differentially expressed (p<0.05, fold >1.5). Panc1: 890 genes differentially expressed (p<0.05, fold >1.5). Common genes: 50.
(DOCX)

S1 Fig Expression and pro-oncogenic functions of MALAT-1. (A) MALAT-1 expression was determined by real time PCR in multiple cancer cell lines and a non-transformed pancreatic cell line (HPDE). Knockdown of MALAT-1 inhibits Panc1 cells using the Ibidi (B) and Boyden chamber (C) assays.
(PDF)
