# Supplementary material for: Role of metastasis-associated lung adenocarcinoma transcript-1 (MALAT-1) in pancreatic cancer
Source: PLoS One. 2018 Feb 1;13(2):e0192264. doi: 10.1371/journal.pone.0192264 (PMC5794178; doi:10.1371/journal.pone.0192264)
Supplement: S1 Table — (DOCX) [file pone.0192264.s001.docx]

**S1 Table. Primers for siRNA studies.**

| Name | Sequence |
| --- | --- |
| SiGL2 | Sigma CGUACGCGGAAUACUUCGA |
| SiMALAT-1#1 | Sigma SASI_Hs02_00377093 |
| SiMALAT-1#2 | Ambion cat#4390771 |
| SiAPAF1 | Sigma SASI_Hs02_00331274 |
| SiEZH2 | Sigma SASI_Hs01_00147882 |
| SiLSD1 | Sigma SASI_Hs01_00213078 |
| SiMLL-1 | Sigma SASI_Hs01_00090459 |
| SiNDRG1 | Sigma SASI_Hs01_00034470 |
| SiSP1 | Sigma SASI_Hs02_00333289 |
| SiSP3 | Sigma SASI_Hs01_00211941 |
| SiSP4 | Sigma SASI_Hs01_00114420 |
