# Supplementary material for: Role of metastasis-associated lung adenocarcinoma transcript-1 (MALAT-1) in pancreatic cancer
Source: PLoS One. 2018 Feb 1;13(2):e0192264. doi: 10.1371/journal.pone.0192264 (PMC5794178; doi:10.1371/journal.pone.0192264)
Supplement: S2 Table — (DOCX) [file pone.0192264.s002.docx]

**S2 Table. Human primers used for real time-PCR.**

| Gene Name | Forward Primer | Reverse Primer |
| --- | --- | --- |
| NRAS | GCACCATAGGTACATCATCCG | GCTTCCTCTGTGTATTTGCCA |
| PCNA | AAGAGAGTGGAGTGGCTTTTG | TGTCGATAAAGAGGAGGAAGC |
| SPRY2 | GAAGTGTGGTCACTCCAGCA | TTGCACATCGCAGAAAGAAG |
| SMAD3 | CGGCAGTAGATGACATGAGG | TCAACACCAAGTGCATCACC |
| APAF1 | CCTCTCATTTGCTGATGTCG | TCACTGCAGATTTTCACCAGA |
| CD44 | TGCTACCAGAGACCAAGACA | CCCATGTGAGTGTCCATCTG |
