# Supplementary material for: Role of metastasis-associated lung adenocarcinoma transcript-1 (MALAT-1) in pancreatic cancer
Source: PLoS One. 2018 Feb 1;13(2):e0192264. doi: 10.1371/journal.pone.0192264 (PMC5794178; doi:10.1371/journal.pone.0192264)
Supplement: S3 Table — (DOCX) [file pone.0192264.s003.docx]

**S3 Table. Mouse primers used for real time-PCR.**

| Gene Name | Forward Primer | Reverse Primer |
| --- | --- | --- |
| PCNA | GGAGACAGTGGAGTGGCTTT | TGGATAAAGAAGAGGAGGCG |
| MALAT-1 | TGAAAAAGGAAATGAGGAGAAAAG | CTTCACAAAACCTCCCTTTACAAT |
| GAPDH | TTGATGGCAACAATCTCCAC | CGTCCCGTAGACAAAATGGT |
