# Supplementary material for: Role of metastasis-associated lung adenocarcinoma transcript-1 (MALAT-1) in pancreatic cancer
Source: PLoS One. 2018 Feb 1;13(2):e0192264. doi: 10.1371/journal.pone.0192264 (PMC5794178; doi:10.1371/journal.pone.0192264)
Supplement: S4 Table — Mouse: 1251 genes differentially expressed (p<0.05, fold >1.5). Panc1: 890 genes differentially expressed (p<0.05, fold >1.5). Common genes: 50. (DOCX) [file pone.0192264.s004.docx]

**S4 Table. The comparison of differentially expressed gene in Panc1 cells and Malat1 transgenic mouse tumors.** Mouse: 1251 genes differentially expressed (p<0.05, fold >1.5). Panc1: 890 genes differentially expressed (p<0.05, fold >1.5). Common genes: 50.

| **Symbol** | **Exp Fold Change(mouse)** | **Exp Fold Change(human)** |
| --- | --- | --- |
| WISP2 | -8.886 | -1.653 |
| TBX2 | -7.193 | -3.774 |
| CPXM1 | -6.869 | -1.858 |
| OLFM1 | -6.733 | -1.861 |
| BCAS1 | -6.492 | -1.531 |
| MDK | -5.778 | -2.014 |
| S1PR3 | -4.863 | -1.652 |
| IGFBP5 | -4.319 | -2.924 |
| TFF1 | -4.250 | -16.009 |
| MYLIP | -4.073 | -1.508 |
| TSPAN6 | -4.024 | -1.903 |
| FOXA1 | -3.961 | -6.948 |
| PPM1H | -3.867 | -1.786 |
| FAM46A | -3.863 | -3.324 |
| TBC1D9 | -3.717 | -1.849 |
| TACSTD2 | -3.599 | -13.771 |
| ASS1 | -3.285 | -1.573 |
| INHBB | -3.225 | -1.646 |
| LHFPL2 | -3.173 | -1.523 |
| TSKU | -3.122 | -4.661 |
| NOTCH3 | -3.117 | -2.204 |
| FAM174B | -3.022 | -2.162 |
| STAT1 | -2.992 | -1.514 |
| LYN | -2.910 | -1.530 |
| UNC93B1 | -2.897 | -1.537 |
| SLC7A2 | -2.821 | -3.742 |
| PARP14 | -2.759 | -1.786 |
| CEBPA | -2.744 | -1.613 |
| DKK3 | -2.669 | -2.378 |
| ALDH1A3 | -2.528 | -1.624 |
| SERPINA3 | -2.508 | -4.231 |
| MAL2 | -2.465 | -4.240 |
| PMEPA1 | -2.448 | -1.502 |
| TAGLN | -2.432 | -1.843 |
| GPD1L | -2.223 | -2.938 |
| NCBP2 | -2.210 | -1.570 |
| MYD88 | -2.149 | -1.572 |
| TMEM64 | -2.143 | -3.618 |
| RIOK3 | 2.143 | 1.573 |
| PSPH | 2.190 | 1.723 |
| VPS37B | 2.269 | 1.560 |
| LMNA | 2.473 | 1.836 |
| PTBP2 | 2.509 | 1.583 |
| NDRG1 | 2.883 | 2.662 |
| SLC16A3 | 3.034 | 1.642 |
| PHGDH | 3.183 | 1.543 |
| HIST1H1C | 3.238 | 1.707 |
| BAG2 | 3.335 | 1.860 |
| CENPM | 3.388 | 1.556 |
