# Supplementary material for: Role of metastasis-associated lung adenocarcinoma transcript-1 (MALAT-1) in pancreatic cancer
Source: PLoS One. 2018 Feb 1;13(2):e0192264. doi: 10.1371/journal.pone.0192264 (PMC5794178; doi:10.1371/journal.pone.0192264)
Supplement: S1 Fig — (A) MALAT-1 expression was determined by real time PCR in multiple cancer cell lines and a non-transformed pancreatic cell line (HPDE). Knockdown of MALAT-1 inhibits Panc1 cells using the Ibidi (B) and Boyden chamber (C) assays. (PDF) [file pone.0192264.s005.pdf]

S1 Figure

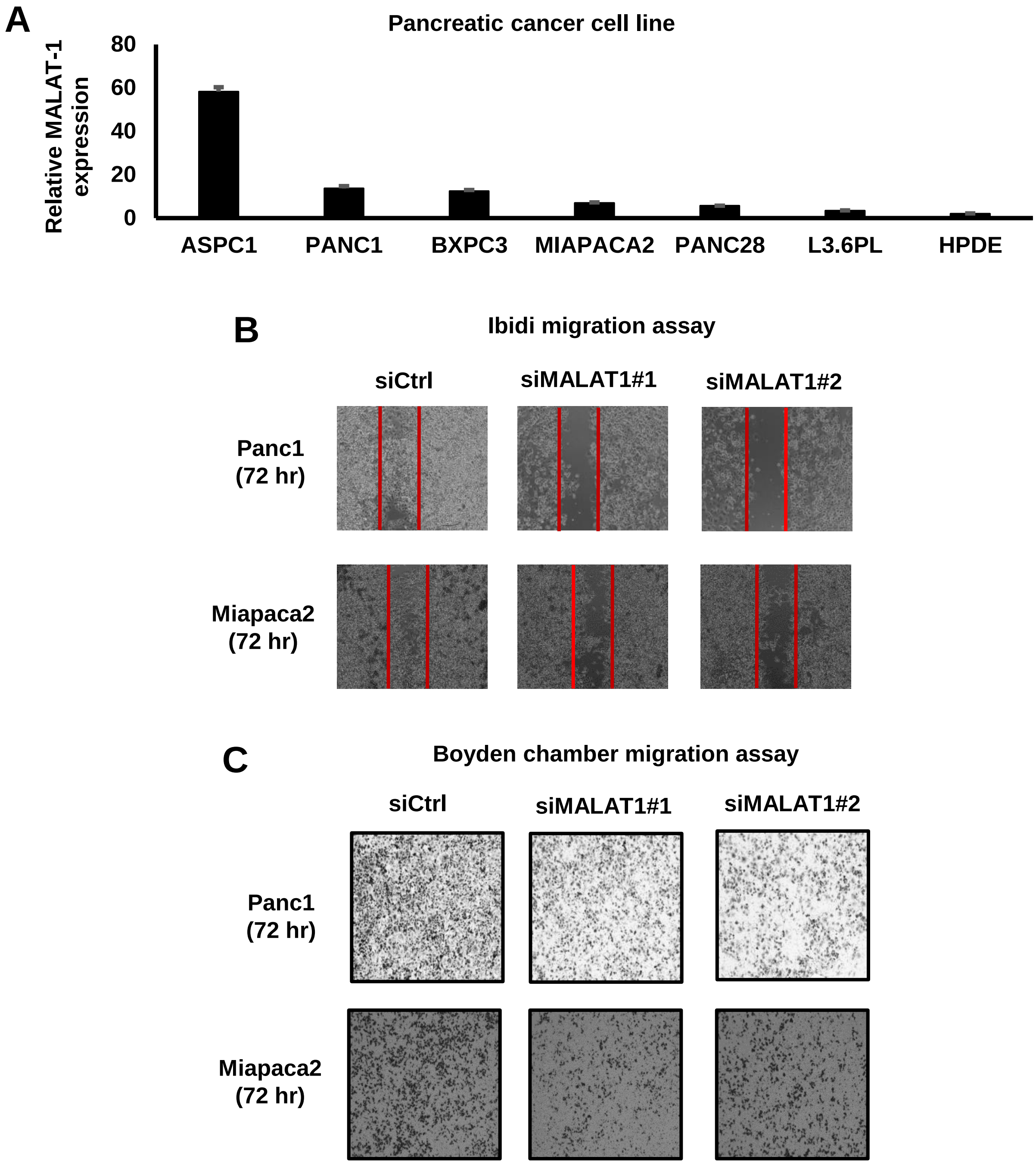

S1 Figure. Expression and pro-oncogenic functions of MALAT-1. (A) MALAT-1 expression was determined by real time PCR in multiple cancer cell lines and a non-transformed pancreatic cell line (HPDE). Knockdown of MALAT-1 inhibits Panc1 cells using the Ibidi (B) and Boyden chamber (C) assays.
